# Supplementary material for: BAP1 constrains pervasive H2AK119ub1 to control the transcriptional potential of the genome
Source: Genes Dev. 2021 May 1;35(9-10):749–70. doi: 10.1101/gad.347005.120 (PMC8091973; doi:10.1101/gad.347005.120)
Supplement: Supplemental Material [file supp_35_9-10_749__index.html]

BAP1 constrains pervasive H2AK119ub1 to control the transcriptional potential of the genome — Supplemental Material 

# BAP1 constrains pervasive H2AK119ub1 to control the transcriptional potential of the genome

## Supplemental Material

- Supplemental\_Material\_Fursova2021.pdf
- Supplemental\_Table\_S1.xlsx
- Supplemental\_Table\_S2.xlsx
- Supplemental\_Table\_S3.xlsx
