# Supplementary material for: BAP1 constrains pervasive H2AK119ub1 to control the transcriptional potential of the genome
Source: Genes Dev. 2021 May 1;35(9-10):749–70. doi: 10.1101/gad.347005.120 (PMC8091973; doi:10.1101/gad.347005.120)
Supplement: Supplemental Material [file supp_gad.347005.120_Supplemental_Material_Fursova2021.pdf]

Supplemental Material

BAP1 constrains pervasive H2AK119ub1 to control the transcriptional potential of the genome

Nadezda A. Fursova, Anne H. Turberfield, Neil P. Blackledge, Emma L. Findlater, Anna Lastuvkova, Miles K. Huseyin, Paula Dobrinić, and Robert J. Klose

Supplemental Figures

Supplemental Figure S1, related to Figure 1. BAP1 functions pervasively throughout the genome to constrain H2AK119ub1.

Supplemental Figure S2, related to Figure 1. BAP1 functions pervasively throughout the genome to constrain H2AK119ub1.

Supplemental Figure S3, related to Figure 2. Pervasive accumulation of H2AK119ub1 in the absence of BAP1 causes widespread reductions in gene expression.

Supplemental Figure S4, related to Figure 3. BAP1 counteracts pervasive H2AK119ub1 to promote Ser5 phosphorylation on the CTD of RNA Pol II at gene regulatory elements.

Supplemental Figure S5, related to Figure 4. Aberrant accumulation of H2AK119ub1 compromises transcription-associated histone modifications but not chromatin accessibility at gene regulatory elements.

Supplemental Figure S6, related to Figure 4. Aberrant accumulation of H2AK119ub1 compromises transcription-associated histone modifications but not chromatin accessibility at gene regulatory elements.

Supplemental Figure S7, related to Figure 5. BAP1 indirectly supports repression of a subset of Polycomb target genes by counteracting pervasive H2AK119ub1 to focus Polycomb complex occupancy at target sites.

Supplemental Tables

Supplemental Table S1, related to Materials and Methods. A list of antibodies used in this study for western blot and cChIP-seq analysis.

Supplemental Table S2, related to Materials and Methods. A list of all next-generation sequencing experiments performed in this study detailing the number of uniquely aligned reads for mouse and spike-in genomes.

Supplemental Table S3, related to Materials and Methods. Summary of pairwise correlations between individual biological replicates for all next-generation sequencing experiments performed in this study.

Supplemental Fig. S1

(A)

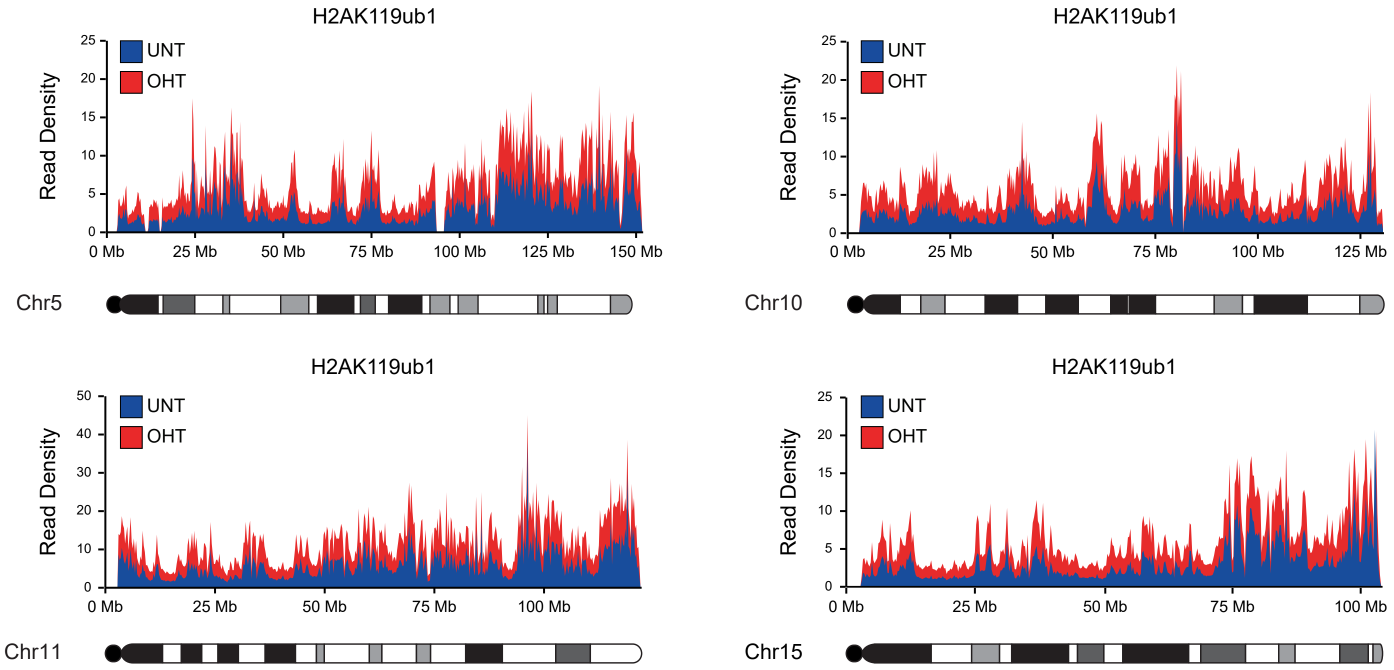

Supplemental Figure S1. Related to Figure 1.

(A) Chromosome density plots showing H2AK119ub1 cChIP-seq signal across chromosomes 5, 10, 11 and 15 in *Bap1*$^{fl/fl}$ ESCs (untreated and OHT-treated). This illustrates a widespread increase in H2AK119ub1 throughout the genome following BAP1 removal.

Supplemental Fig. S2

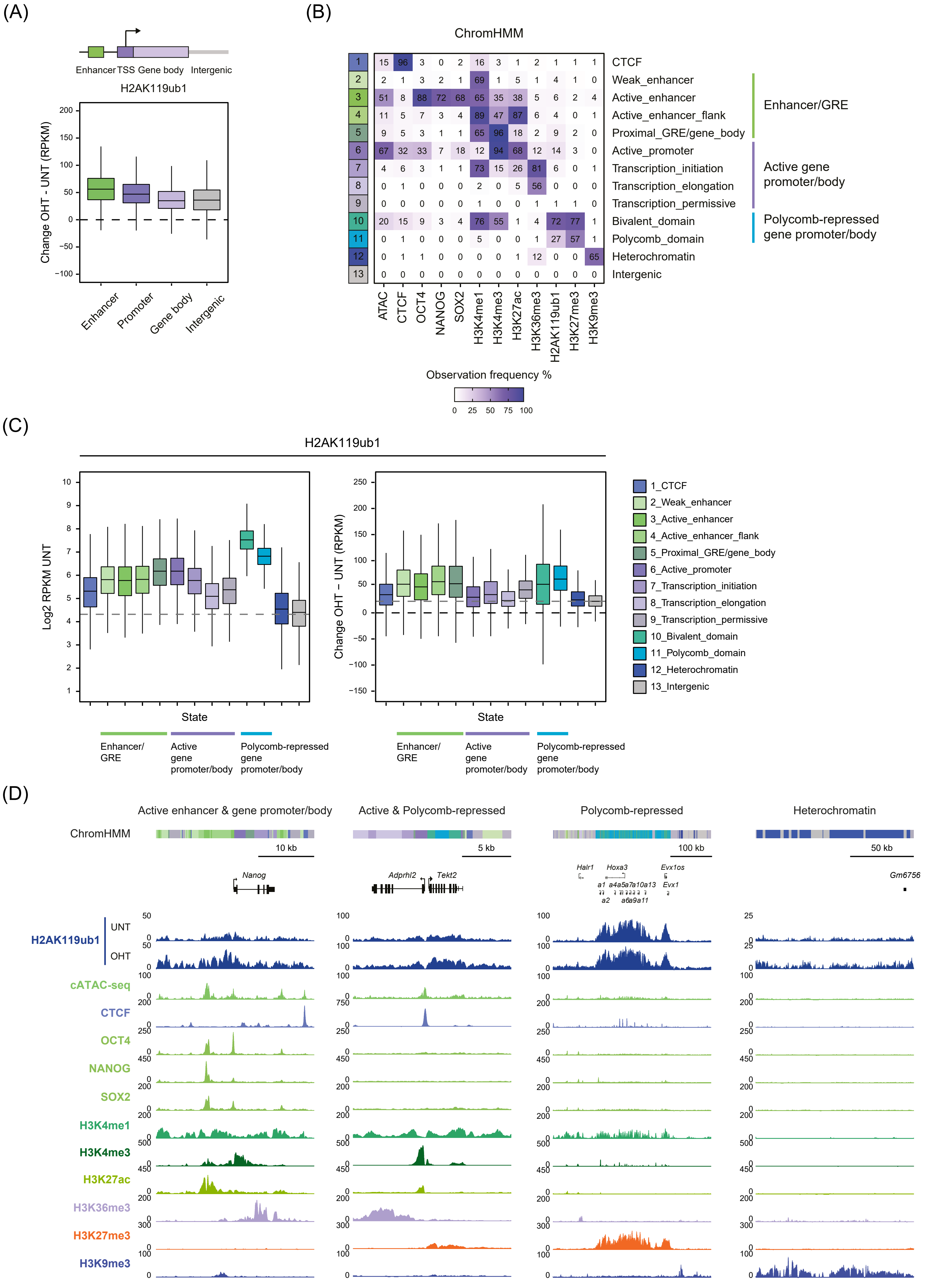

Supplemental Figure S2. Related to Figure 1.

- **(A)** Boxplots showing the absolute change in H2AK119ub1 cChIP-seq signal at gene regulatory elements (enhancers and promoters), gene bodies, and intergenic regions following OHT treatment in *Bap1*$^{fl/fl}$ ESCs.
- **(B)** A heatmap summarizing emission parameters for the ChromHMM model used to segment the genome into 13 chromatin states. Each row of the heatmap corresponds to one of the chromatin states, which are color-coded and grouped based on the underlying gene regulatory elements (GREs) and transcriptional activity. Columns correspond to different chromatin features that were used to build the model. The heatmap color intensity reflects the probability of observing a particular chromatin feature in the specific chromatin state.
- **(C)** Boxplots comparing H2AK119ub1 cChIP-seq signal in untreated *Bap1*$^{fl/fl}$ ESCs (*left panel*), as well as the absolute change in this signal following OHT treatment in *Bap1*$^{fl/fl}$ ESCs *(right panel)*, across different chromatin states defined by the ChromHMM model in (B). The dashed grey line represents the average levels of H2AK119ub1 in untreated cells (*left panel*) or the average change in H2AK119ub1 following BAP1 removal *(right panel)* across the genome, as determined by their median values in intergenic regions.
- **(D)** Snapshots of genomic regions encompassing different chromatin states defined by ChromHMM. ChIP-seq tracks for all chromatin features that were used to build the ChromHMM model in (B) are shown together with H2AK119ub1 cChIP-seq in untreated and OHT-treated *Bap1*$^{fl/fl}$ ESCs. The segmentation of genomic regions into chromatin states is illustrated by the colored bar at the top of each panel, with different states represented by the same colors as in (B).

Supplemental Fig. S3

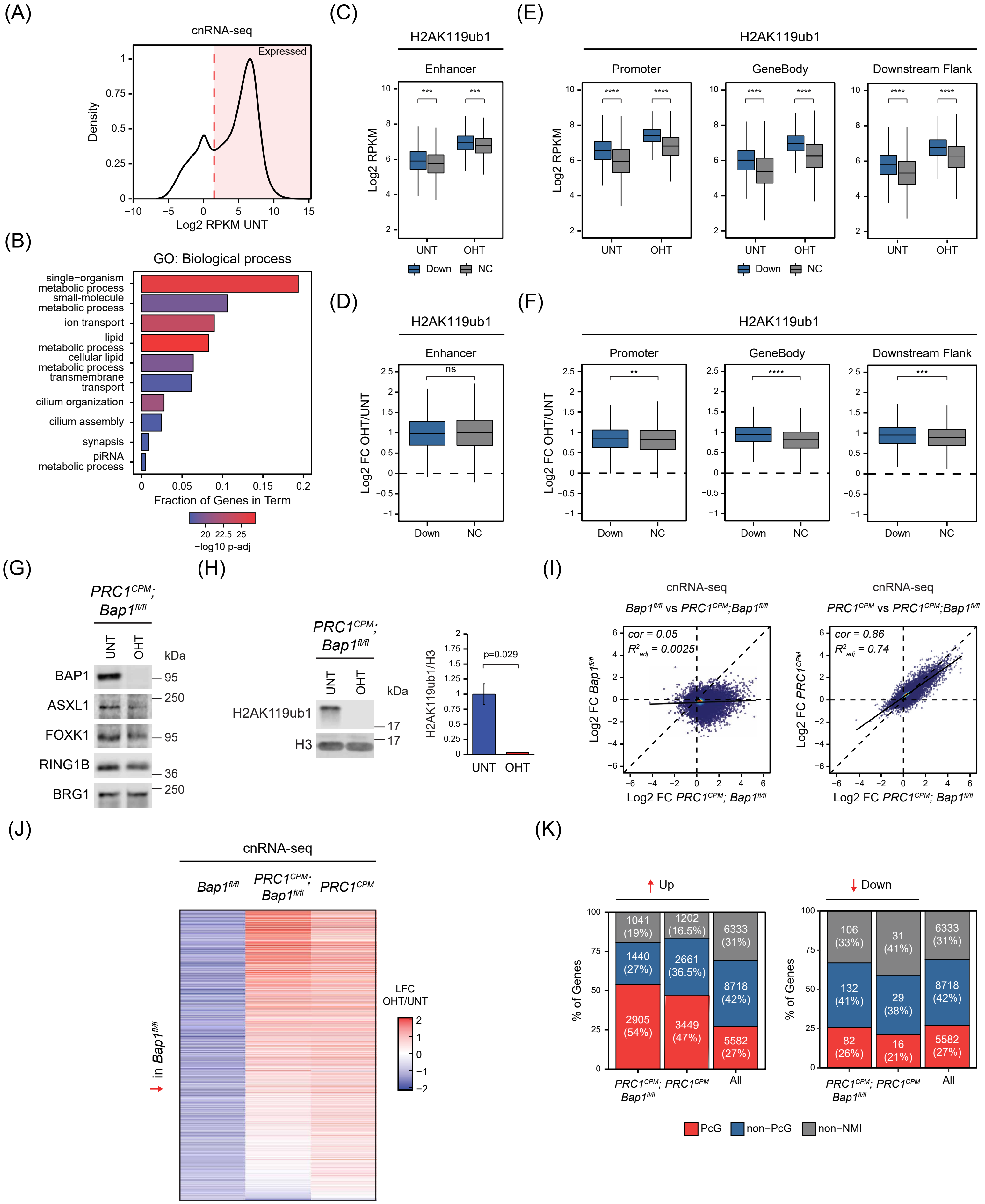

Supplemental Figure S3. Related to Figure 2.

- **(A)** Density plot of log2-transformed RPKM gene expression levels (cnRNA-seq) in untreated *Bap1$^{fl/fl}$* ESCs. The red line indicates the cutoff (RPKM = 2.838) that was used to separate robustly expressed genes (n = 14,779) from genes with no or very low expression.
- **(B)** A gene ontology (GO) analysis of Biological Process term enrichment for genes showing a significant reduction in expression (p-adj < 0.05 and > 1.5-fold) in *Bap1$^{fl/fl}$* cells following OHT treatment.
- **(C)** Boxplots comparing H2AK119ub1 cChIP-seq signal in *Bap1$^{fl/fl}$* ESCs (untreated and OHT-treated) at the nearest putative enhancers associated with genes that show a significant reduction (Down, n = 1916) or no change (No Change, n = 9647) in expression following BAP1 removal based on cnRNA-seq analysis (p-adj < 0.05 and > 1.5-fold). P-values denote the result of a two-tailed Student's t-test: (***) $P < 10^{-10}$.
- **(D)** Boxplots comparing log2-fold changes in H2AK119ub1 cChIP-seq signal following OHT treatment in *Bap1$^{fl/fl}$* ESCs at the nearest putative enhancers associated with genes that were split into two groups as defined in (C). P-values denote the result of a two-tailed Student's t-test: (ns) P > 0.05.
- **(E)** Boxplots comparing H2AK119ub1 cChIP-seq signal in *Bap1$^{fl/fl}$* ESCs (untreated and OHT-treated) at the promoters, bodies and 10 kb downstream flanking regions of genes that show a significant reduction (Down, n = 2828) or no change (No Change, n = 17,203) in expression following BAP1 removal based on cnRNA-seq analysis (p-adj < 0.05 and > 1.5-fold). P-values denote the result of a two-tailed Student's t-test: (****) $P < 10^{-100}$.
- **(F)** Boxplots comparing log2-fold changes in H2AK119ub1 cChIP-seq signal following OHT treatment in *Bap1$^{fl/fl}$* ESCs at the promoters, bodies and 10 kb downstream flanking regions of genes that were split into two groups as defined in (E). P-values denote the result of a two-tailed Student's t-test: (****) $P < 10^{-100}$, (***) $P < 10^{-10}$, (**) $P < 10^{-5}$.
- **(G)** Western blot analysis for the PR-DUB complex subunits (BAP1, ASXL1 and FOXK1) and the PRC1 catalytic subunit RING1B in untreated and OHT-treated *PRC1^{CPM};Bap1$^{fl/fl}$* ESCs. BRG1 is shown as a loading control.
- **(H)** Western blot analysis (*left panel*) and quantification (*right panel*) of H2AK119ub1 levels relative to histone H3 in untreated and OHT-treated *PRC1^{CPM};Bap1$^{fl/fl}$* ESCs. Error bars represent SEM (n = 3). P-value denotes the result of a paired two-tailed Student's t-test.
- **(I)** Scatterplots comparing the log2-fold changes in gene expression (cnRNA-seq) following OHT treatment in *Bap1$^{fl/fl}$* and *PRC1^{CPM};Bap1$^{fl/fl}$* ESCs (*left panel*), as well as *PRC1^{CPM}* and *PRC1^{CPM};Bap1$^{fl/fl}$* ESCs (*right panel*). R^2_{adj} represents the adjusted coefficient of determination

for linear regression, and *cor* denotes the Pearson correlation coefficient. This illustrates that simultaneous removal of BAP1 and catalytic activity of PRC1 closely recapitulates the gene expression defects manifesting from disrupting PRC1 catalysis alone, revealing an epistatic genetic interaction between PRC1 (H2AK119ub1) and BAP1.

(J) A heatmap illustrating log2-fold changes in expression (cnRNA-seq) following OHT treatment (LFC OHT/UNT) in *Bap1*$^{fl/fl}$, *PRC1*^{CPM}*;Bap1*$^{fl/fl}$ and *PRC1*^{CPM} ESCs for genes that show a significant reduction in expression (p-adj < 0.05 and > 1.5-fold) after BAP1 removal.

(K) Bar plots illustrating the distribution of different gene classes among genes that show significant increases (*left*) or decreases (*right*) in expression following OHT treatment in *PRC1*^{CPM}*;Bap1*$^{fl/fl}$ and *PRC1*^{CPM} ESCs based on cnRNA-seq analysis (p-adj < 0.05 and > 1.5-fold). PcG corresponds to Polycomb-occupied genes; Non-PcG to non-Polycomb-occupied genes; Non-NMI to genes lacking a non-methylated CGI (NMI) at their promoter.

Supplemental Fig. S4

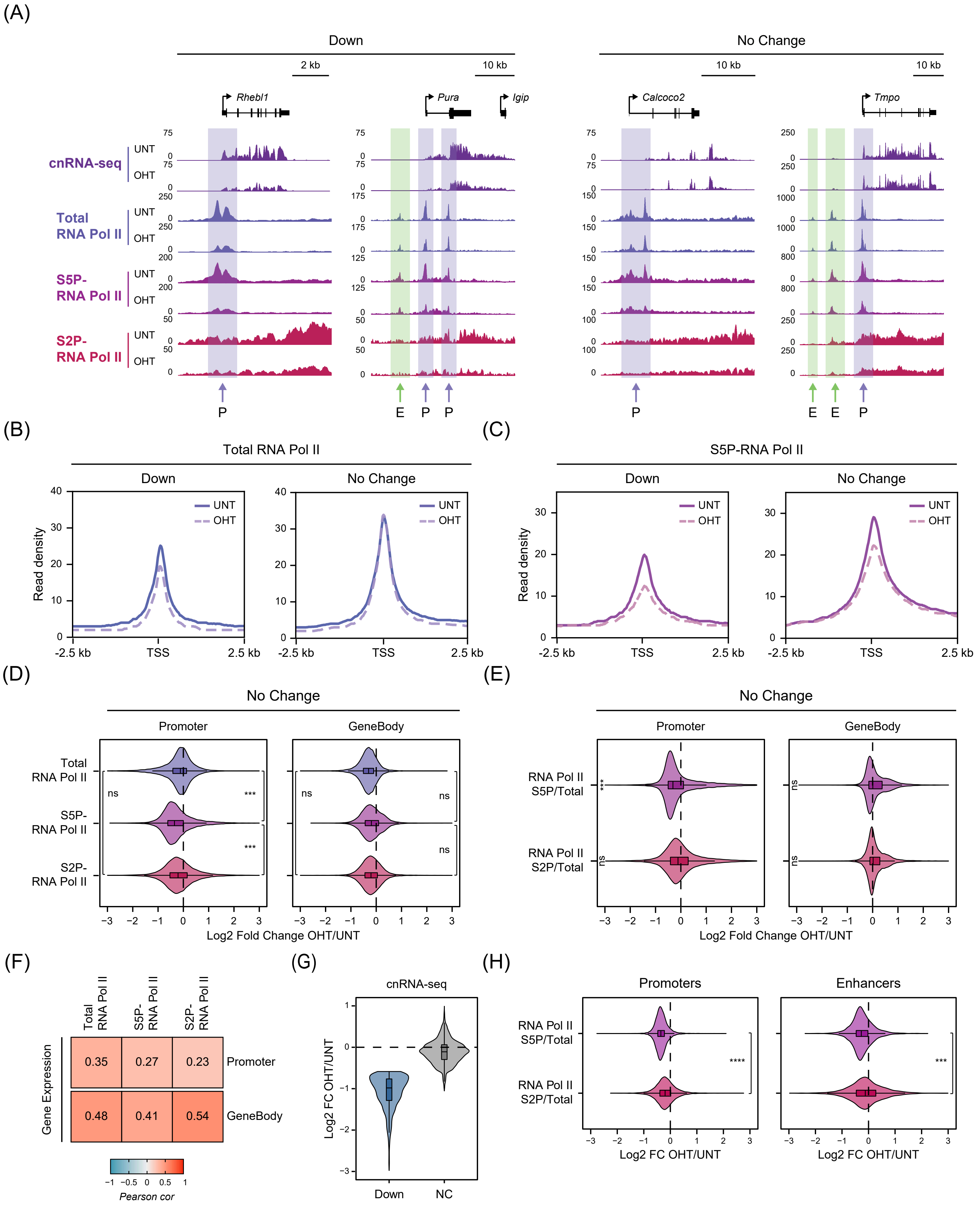

Supplemental Figure S4. Related to Figure 3.

(A) Snapshots of genes that display a significant reduction (Down) or no change in expression following removal of BAP1 based on cnRNA-seq analysis (p-adj < 0.05 and > 1.5-fold). Gene expression (cnRNA-seq) and cChIP-seq for total Pol II, as well as its Ser5- and Ser2-phosphorylation (S5P- and S2P-Pol II), are shown in *Bap1*$^{fl/fl}$ ESCs (untreated and OHT-treated). Positions of promoters (P) (H3K27ac-high, H3K4me3-high) and the nearest putative enhancers (E) (H3K27ac-high, H3K4me3-low) for these genes are indicated (also see Figure S5A).

(B) Metaplots of total Pol II cChIP-seq signal in *Bap1*$^{fl/fl}$ ESCs (untreated and OHT-treated) at the promoters of genes that show a significant reduction (Down, n = 2828) or no change (No Change, n = 17,203) in expression after BAP1 removal based on cnRNA-seq analysis (p-adj < 0.05 and > 1.5-fold).

(C) As in (B) but for Ser5P Pol II cChIP-seq signal.

(D) Violinplots comparing log2-fold changes in cChIP-seq signal for total Pol II, as well as its Ser5P and Ser2P forms, following OHT treatment in *Bap1*$^{fl/fl}$ ESCs at the promoters and over the bodies of genes that show no significant change (No Change, n = 17,203) in expression after BAP1 removal based on cnRNA-seq analysis (p-adj < 0.05 and > 1.5-fold). P-values denote the result of a one-tailed Student's t-test: (***) P < 10^{-10}, (ns) P > 0.05. For comparisons of Ser5P/Ser2P with total Pol II, the alternative hypothesis was that the log2-fold change in Ser5P/Ser2P was smaller. For the comparison of Ser5P with Ser2P, the alternative hypothesis was that the log2-fold change in Ser5P was smaller.

(E) Violinplots comparing log2-fold changes in the abundance of Ser5P and Ser2P relative to total Pol II levels (S5P/Total and S2P/Total) following OHT treatment in *Bap1*$^{fl/fl}$ ESCs at the promoters and over the bodies of genes defined in (D). P-values denote the result of a one-sample one-tailed Student's t-test to determine whether the log2-fold changes were significantly smaller than 0: (***) P < 10^{-10}, (ns) P > 0.05.

(F) Correlation of log2-fold changes in gene expression (cnRNA-seq) with log2-fold changes in cChIP-seq signal for total Pol II, as well as its Ser5- and Ser2-phosphorylation (S5P- and S2P-Pol II), at gene promoters and bodies in *Bap1*$^{fl/fl}$ cells following OHT treatment.

(G) Violinplots comparing log2-fold changes in gene expression (cnRNA-seq) following OHT treatment in *Bap1*$^{fl/fl}$ ESCs for genes that show a significant reduction (Down, n = 2828) or no change (No Change, n = 17,203) in expression after BAP1 removal based on cnRNA-seq analysis (p-adj < 0.05 and > 1.5-fold). This illustrates that expression of genes that are not classified as showing significant changes is still reduced following BAP1 removal, albeit very modestly.

(H) Violinplots comparing log2-fold changes in the abundance of Ser5P and Ser2P relative to total Pol II levels (S5P/Total and S2P/Total) following OHT treatment in *Bap1*$^{fl/fl}$ ESCs at active gene promoters and enhancers. P-values denote the result of a one-tailed Student's t-test with the alternative hypothesis that the log2-fold change in Ser5P/total Pol II ratio was smaller: (****) $P < 10^{-100}$, (***) $P < 10^{-10}$.

Supplemental Fig. S5

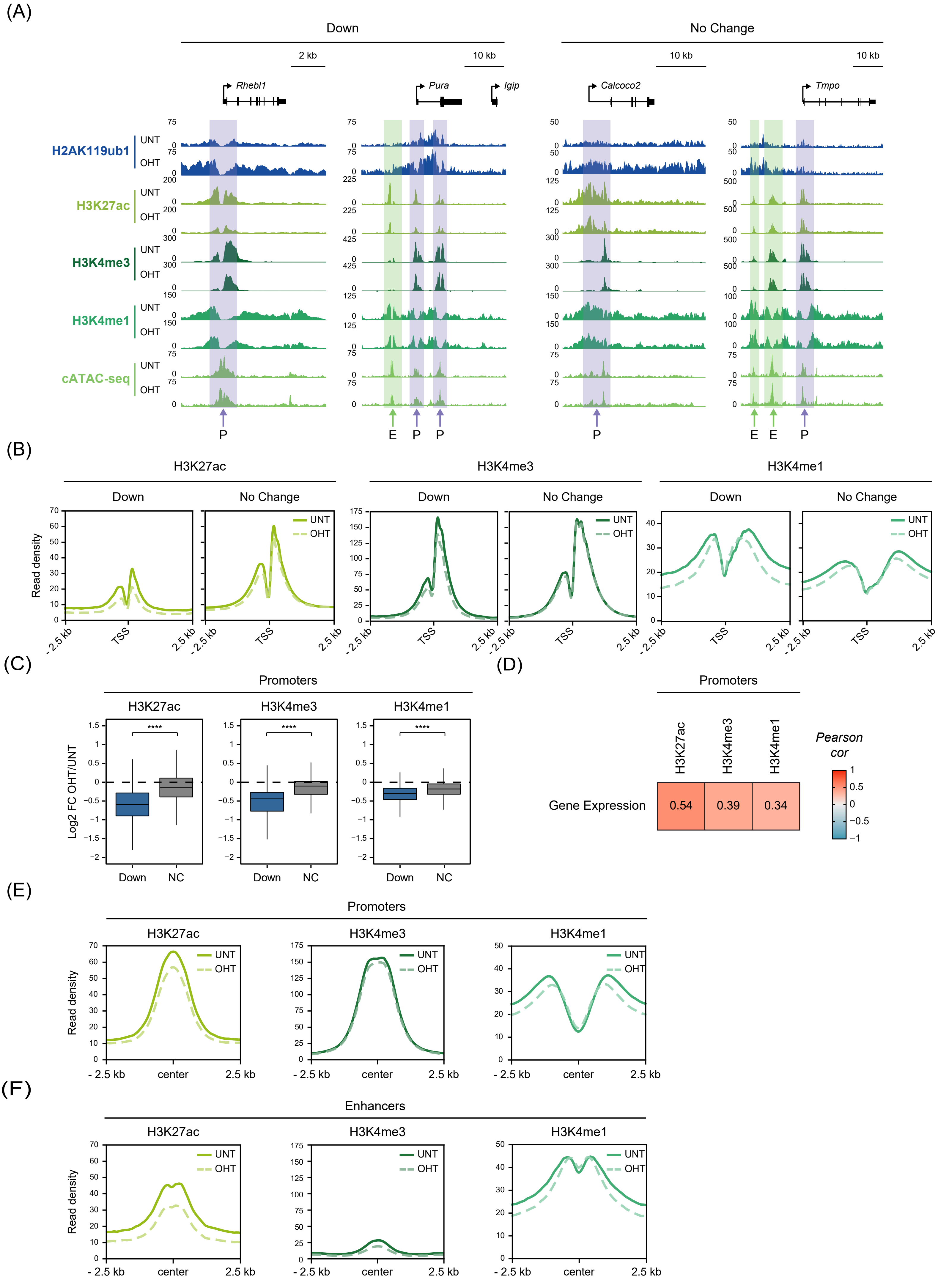

Supplemental Figure S5. Related to Figure 4.

(A) Snapshots of genes that display a significant reduction (Down) or no change in expression following removal of BAP1 based on cnRNA-seq analysis (p-adj < 0.05 and > 1.5-fold). cChIP-seq is shown for H2AK119ub1, H3K27ac, H3K4me3 and H3K4me1 in *Bap1*$^{fl/fl}$ ESCs (untreated and OHT-treated). cATAC-seq is also shown as a measure of chromatin accessibility. Positions of promoters (P) (H3K27ac-high, H3K4me3-high) and the nearest putative enhancers (E) (H3K27ac-high, H3K4me3-low) for these genes are indicated.

(B) Metaplots illustrating H3K27ac, H3K4me3 and H3K4me1 cChIP-seq signal in *Bap1*$^{fl/fl}$ ESCs (untreated and OHT-treated) at the promoters of genes that show a significant reduction (Down, n = 2828) or no change (No Change, n = 17,203) in expression after BAP1 removal based on cnRNA-seq analysis (p-adj < 0.05 and > 1.5-fold).

(C) Boxplots comparing log2-fold changes in H3K27ac, H3K4me3 and H3K4me1 cChIP-seq signal following OHT treatment in *Bap1*$^{fl/fl}$ ESCs at the promoters of genes described in (B). P-values denote the result of a two-tailed Student's t-test: (****) $P < 10^{-100}$.

(D) Correlation of log2-fold changes in gene expression (cnRNA-seq) with log2-fold changes in cChIP-seq signal for H3K27ac, H3K4me3 and H3K4me1 at gene promoters following OHT treatment in *Bap1*$^{fl/fl}$ cells.

(E) Metaplots illustrating H3K27ac, H3K4me3 and H3K4me1 cChIP-seq signal at active gene promoters in *Bap1*$^{fl/fl}$ ESCs (untreated and OHT-treated).

(F) As in (E) but for active enhancers.

Supplemental Fig. S6

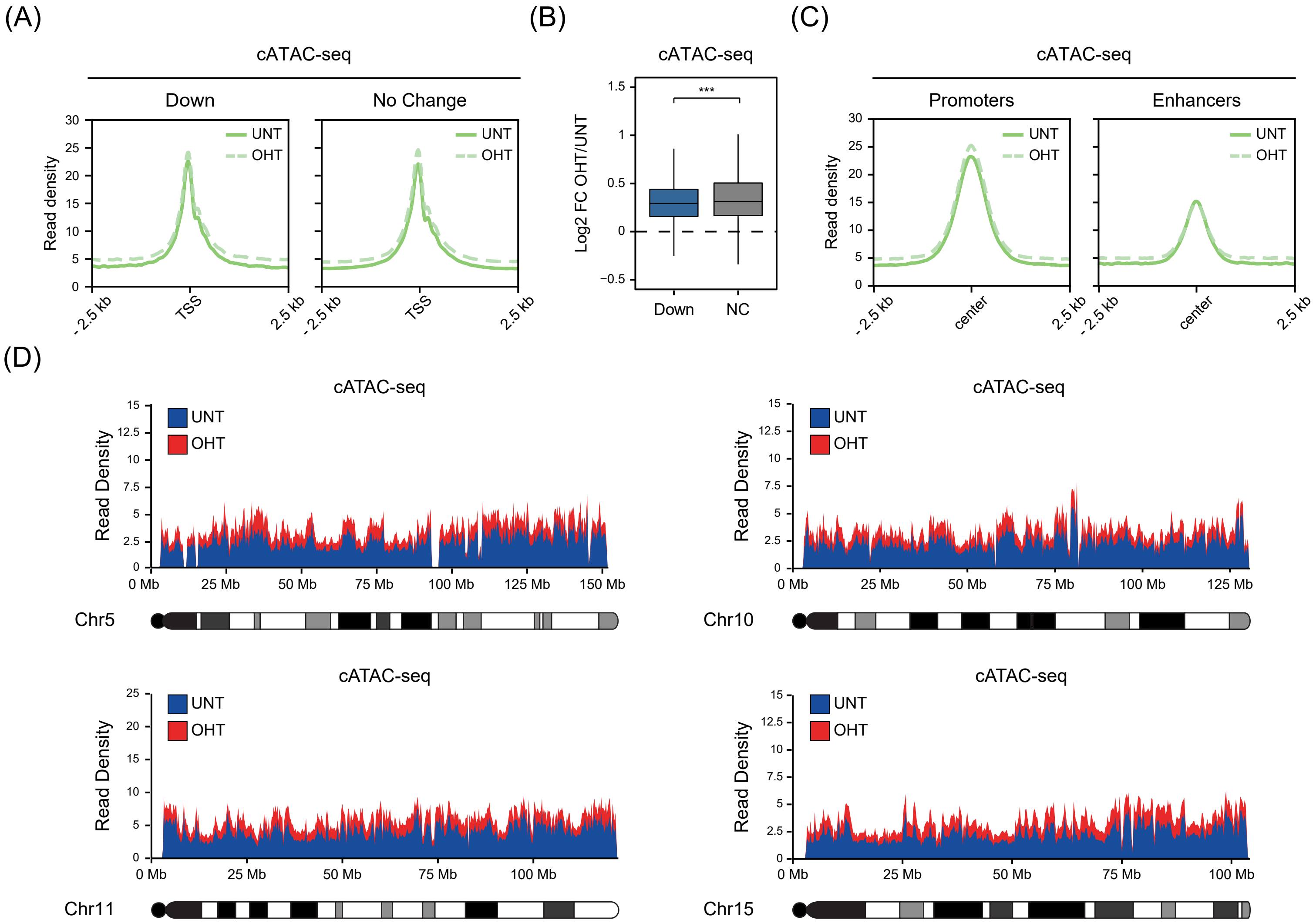

Supplemental Figure S6. Related to Figure 4.

(A) Metaplots illustrating cATAC-seq signal in *Bap1*$^{fl/fl}$ ESCs (untreated and OHT-treated) at the promoters of genes that show a significant reduction (Down, n = 2828) or no change (No Change, n = 17,203) in expression after BAP1 removal based on cnRNA-seq analysis (p-adj < 0.05 and > 1.5-fold).

(B) Boxplots comparing log2-fold changes in cATAC-seq signal following OHT treatment in *Bap1*$^{fl/fl}$ ESCs at the promoters of genes described in (A). P-value denotes the result of a two-tailed Student's t-test: (***) $P < 10^{-10}$.

(C) Metaplots illustrating cATAC-seq signal at active gene promoters (*left*) and active enhancers (*right*) in *Bap1*$^{fl/fl}$ ESCs (untreated and OHT-treated).

(D) Chromosome density plots showing chromatin accessibility as measured by cATAC-seq across chromosomes 5, 10, 11 and 15 in *Bap1*$^{fl/fl}$ ESCs (untreated and OHT-treated). This illustrates a modest yet widespread increase in cATAC-seq signal throughout the genome following BAP1 removal.

Supplemental Fig. S7

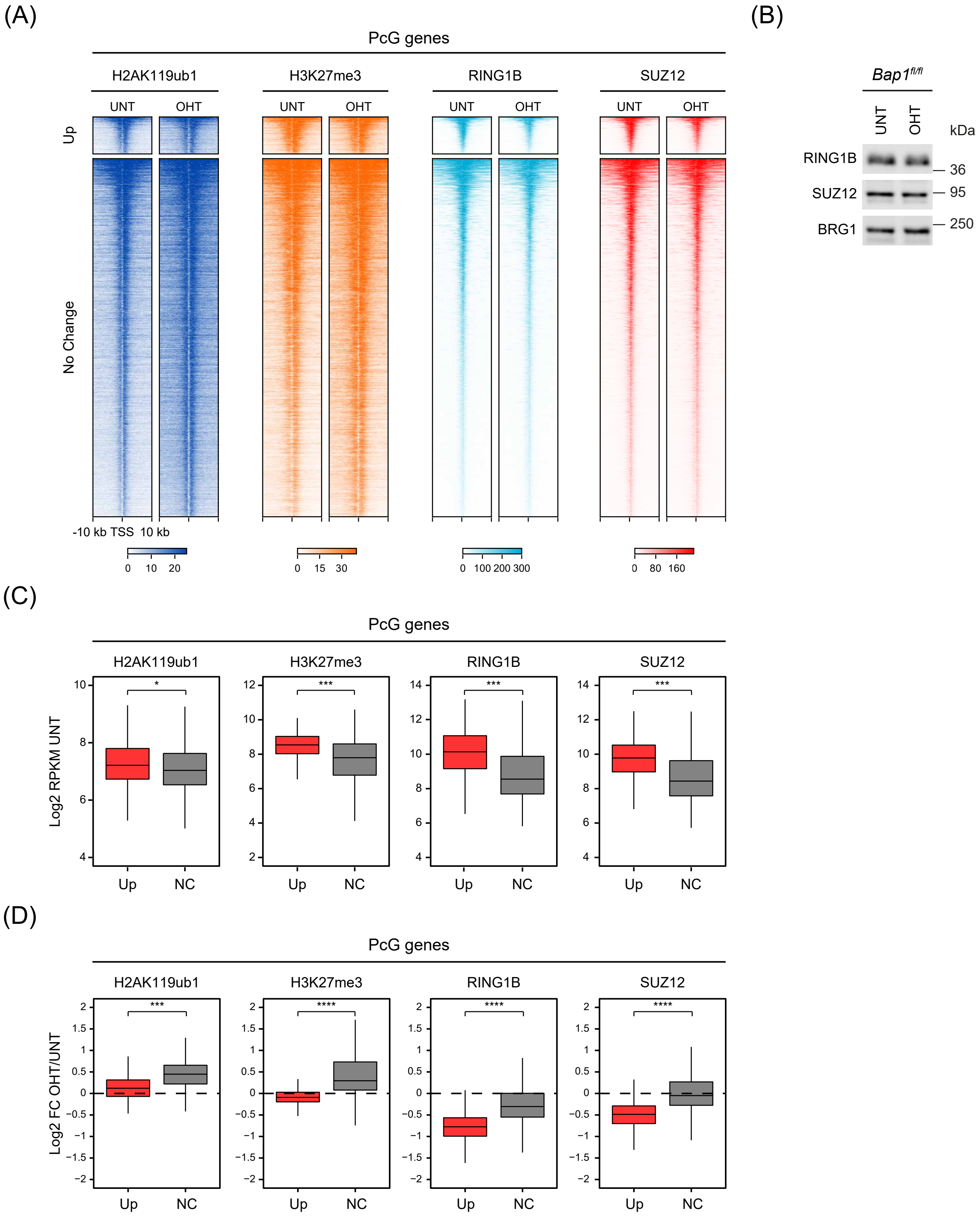

Supplemental Figure S7. Related to Figure 5.

(A) Heatmaps of cChIP-seq signal for H2AK119ub1, H3K27me3, RING1B (PRC1) and SUZ12 (PRC2) in *Bap1*$^{fl/fl}$ ESCs (untreated and OHT-treated) at the promoters of Polycomb-occupied genes that become significantly derepressed (Up, n = 421) or do not change in expression (No Change, n = 4075) following BAP1 removal based on cnRNA-seq analysis (p-adj < 0.05 and > 1.5-fold). Intervals were sorted by RING1B occupancy in untreated *Bap1*$^{fl/fl}$ ESCs.

(B) Western blot analysis for RING1B (PRC1) and SUZ12 (PRC2) in untreated and OHT-treated *Bap1*$^{fl/fl}$ ESCs. BRG1 is shown as a loading control.

(C) Boxplots comparing cChIP-seq signal for H2AK119ub1, H3K27me3, RING1B and SUZ12 in untreated *Bap1*$^{fl/fl}$ ESCs at the promoters of Polycomb-occupied genes that become significantly derepressed (Up, n = 421) or do not change in expression (No Change, n = 4075) following BAP1 removal based on cnRNA-seq analysis (p-adj < 0.05 and > 1.5-fold). P-values denote the result of a two-tailed Student's t-test: (***) $P < 10^{-10}$, (*) $P < 0.05$.

(D) Boxplots comparing log2-fold changes in cChIP-seq signal for H2AK119ub1, H3K27me3, RING1B and SUZ12 following OHT treatment in *Bap1*$^{fl/fl}$ ESCs at the promoters of Polycomb-occupied genes that become significantly derepressed (Up, n = 421) or do not change in expression (No Change, n = 4075) after BAP1 removal. P-values denote the result of a two-tailed Student's t-test: (****) $P < 10^{-100}$, (***) $P < 10^{-10}$.
